# Efficacy and safety of transanal endoscopic microsurgery for early rectal cancer: a meta-analysis

**DOI:** 10.3389/fonc.2025.1545547

**Published:** 2025-02-10

**Authors:** Chunqiang Wang, Tianye Huang, Xuebing Wang

**Affiliations:** ^1^ Affiliated Xiaoshan Hospital, Hangzhou Normal University, Hangzhou, Zhejiang, China; ^2^ The First Medical University of Shandong Affiliated Linyi Hospital, Linyi, Shandong, China; ^3^ The First Medical University of Shandong Affiliated Taian Hospital, Taian, Shandong, China

**Keywords:** early rectal cancer, transanal endoscopic microsurgery, local excision, endoscopic surgery, safety and efficacy

## Abstract

**Background:**

The gold standard for the treatment of rectal cancer is radical surgery with total mesorectal excision (TME). As one of the alternatives to radical surgery, local resection has been proposed for the treatment of early rectal cancer. The purpose of this article was to evaluate the safety and efficacy of transanal endoscopic microsurgery (TEM) in the treatment of early rectal cancer.

**Methods:**

By searching the PubMed, Cochrane Library, Web of Science, and China National Knowledge Infrastructure databases, we selected all articles on TEM for early rectal cancer. Two researchers independently completed the entire process from screening, inclusion to data extraction and performed statistical analysis using RevMan 5.3. The primary outcomes included basic patient characteristics, overall survival rate, disease-free survival rate, disease-specific survival rate, recurrence rate, and complication rate and type.

**Results:**

A total of 33 articles were included in this meta-analysis. The results showed that the overall survival rate was 100% for T0 stage, 98.1% for Tis (carcinoma *in situ*) stage, and 80.2% for early stage rectal cancer patients (83.9% for T1 and 72.4% for T2). The weighted overall survival rate was 94% (RD = 0.94, 95% CI = 0.93–0.95, *I* ^2^ = 80%, *P* < 0.00001) for all stage patients, the weighted disease-free survival rate was 91% (RD = 0.91, 95% CI = 0.90–0.93, *I* ^2^ = 83%, *P* < 0.00001), and the disease-specific survival rate was 97% (RD = 0.97, 95% CI = 0.96–0.98, *I* ^2^ = 63%, *P* < 0.00001). The recurrence rate was 0.5% for T0 stage, 1.9% for Tis stage, and 11.9% for early stage rectal cancer patients (8.1% for T1 and 19.7% for T2). The weighted recurrence rate was 7% (RD = 0.07, 95% CI = 0.06–0.08, *I* ^2^ = 69%, *P* < 0.00001) for all stage patients. The weighted complications rate was 11% (RD = 0.11, 95% CI = 0.10–0.12, *I* ^2^ = 66%, *P* < 0.00001) for all stage patients, with Clavien-Dindo grade I accounting for 77.7%, Clavien-Dindo grade II accounting for 8%, and Clavien-Dindo grade III accounting for 14.3%.

**Conclusion:**

The results showed that TEM has a high postoperative survival rate, low recurrence rate, and low complication rate in the T0 stage, Tis stage, and T1 stage, indicating its good safety and efficacy. For the treatment of T2 stage, TEM has a lower overall survival rate and a higher recurrence rate. Our meta-analysis results suggest that TEM alone is not recommended as a curative treatment for T2 stage; on the contrary, TME is more frequently recommended.

## 1 Introduction

As one of the common malignancies, the incidence and mortality of colorectal cancer in all cancer diseases have long been at the forefront globally. According to the latest data statistics, there were 1.92 million new cases of colorectal cancer worldwide in 2022, ranking third among all tumors, and 904,000 deaths, ranking second among all tumors. In China, there were about 517,000 new cases of colorectal cancer in 2022, accounting for 26.9% of the world’s new cases of colorectal cancer, and about 240,000 deaths from colorectal cancer, accounting for 26.5% of the world’s colorectal deaths (1–3). A reported cancer-specific survival rate is over 95% at 5 years after radical resection as the gold standard surgical modality for the treatment of early rectal cancer (4, 5). However, although radical surgery is valid in removing the cancer, patient death and local recurrence cannot be completely avoided. Moreover, the occurrence of postoperative complications, such as about 26% of genitourinary dysfunction, 5%–10% of anastomotic fistula, and about 30% of temporary or permanent stomas, severely reduces the quality of life of patients (6–11). Therefore, local resection was proposed as one of the alternatives for the treatment of early rectal cancer. At present, the common clinical local resections include endoscopic submucosal dissection, transanal minimally invasive surgery, standard transanal excision, and so forth (12–14). However, these local resection methods have not been widely accepted due to positive tumor resection margins and increased reports of tumor fragments (15).

With the development of the concept of minimally invasive surgery, Buess et al. proposed a local resection procedure for early rectal cancer in 1983 and named it transanal endoscopic microsurgery (TEM) (16). Some clinical studies have shown that TEM has a lower recurrence rate and complication rate compared with traditional transanal local resection, and postoperative patients have a higher quality of life (17, 18). The emergence and development of TEM provide new surgical options for the treatment of early rectal cancer. Due to its less invasive nature, the local amplification function increases the possibility of resection of complete specimens with lateral and vertical margins without tumor, which is considered the most effective method for complete resection of local tumors (19). Therefore, TEM is considered the preferred local resection method for the treatment of early rectal cancer and is currently used in more than 400 centers worldwide for therapeutic use in patients with T1 or T2 rectal cancer without lymph node metastasis and distant metastases (20, 21). Because there is still about an 8.6% risk of lymph node metastasis in early rectal cancer, and TEM cannot remove lymph nodes, the postoperative recurrence rate and survival rate still need a lot of clinical data research to observe (22). Therefore, there is no unified conclusion on the safety and efficacy of TEM in early rectal cancer.

As far as we know, no one has ever analyzed the safety and efficacy of TEM in early rectal cancer alone. This meta-analysis aims to collect relevant literature and analyze the efficacy and safety of TEM in the treatment of patients with early rectal cancer.

## 2 Materials and methods

### 2.1 Search strategy

According to the PRISMA, we searched the PubMed, Cochrane Library, Web of Science, and China National Knowledge Infrastructure databases (inception-June 2024). We used the following keywords and subheadings: “transanal endoscopic microsurgery,” “rectal cancer,” “rectal tumor,” “local excision,” and “TEM.” In addition, we also manually searched the reference data in the retrieved literature.

### 2.2 Inclusion and exclusion criteria

*Inclusion criteria:* Rectal cancer; the pathological stage was either T1 or T2, and the preoperative examination showed no lymph node metastasis and distant metastasis; the surgical procedure was TEM; clinical research.

*Exclusion criteria:* The patient data was incomplete to extract the required study data; republished literature; randomized controlled trial or meta-analysis; the full text could not be accessed.

### 2.3 Data extraction

Two reviewers individually screened the retrieved literature according to the search criteria, and the controversial article was finally decided by the corresponding author. The extracted information included details of the included studies, tumor stage, postoperative recurrence rate, survival rate, and postoperative complication rate and type.

### 2.4 Risk of bias assessment and quality evaluation

The methodological index for non-randomized studies (MINORS) was adopted to assess risk bias and the quality of the included studies. This tool consists of eight domains (1): aim (2), inclusion criteria (3), exclusion criteria (4), intervention description (5), outcome measures (6), follow-up duration (7), follow-up rate (8), method of statistical analysis. A score of 0 indicates no report at all, 1 indicates reported but incomplete information, and 2 indicates reported and sufficient information. Higher scores indicate less risk bias. It was defined as good (score 11–16), average (score 5–10), or poor (score 0–4).

### 2.5 The procedure of TEM

The rectoscope was inserted into the rectum to the tumor site, and the electric knife made a circular margin marking line along the normal rectal mucosa 1 cm outside the base edge of the tumor. The tumor tissue was completely removed along the marking line. The wound was continuously closed with sutures.

### 2.6 Statistical analysis

We used Revman 5.3 from the Cochrane Collaboration for the statistical analysis. Risk differences (RD) and 95% confidence intervals (CI) are used to represent the dichotomous variables. Based on the heterogeneity of the *I* ^2^ tests, the results were evaluated using a random effect model (*I* ^2^ > 75%) or a fixed effect model (*I* ^2^ < 75%). *P* < 0.05 was considered statistically significant. Potential biases are shown as forest plots.

## 3 Results

### 3.1 Eligible studies

**Figure 1:**
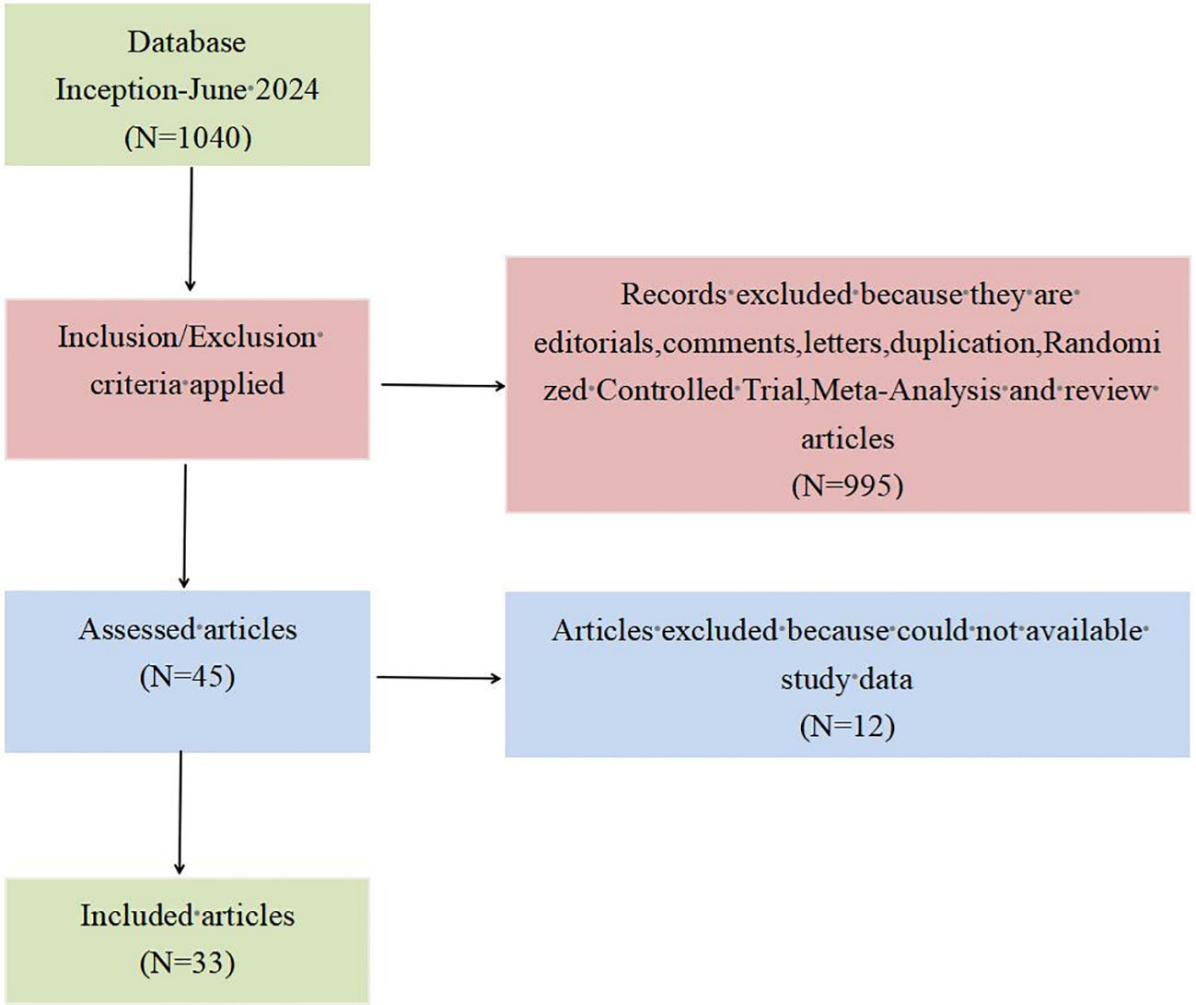
PRISMA flow diagram for the literature search.

By searching the literature in the database, a total of 1,040 related articles on TEM for early rectal cancer were found. After initial screening that followed the inclusion and exclusion criteria, we obtained 45 relevant articles by reading the titles and abstracts. Then, by reading the full text of the initially screened articles, we again excluded articles for which the required study data were not available. Finally, a total of 33 articles (23–55) were obtained and included in this study. The specific PRISMA flowchart is shown in **Figure 1**.

### 3.2 Primary characteristics of included literature

A total of 33 articles published between 1996 and 2024 were included in this meta-analysis. These articles contained a total of 2,160 patients treated with TEM, including 105 Tis stage, 263 T0 stage, 1,208 T1 stage, and 584 T2 stage. In terms of quality evaluation, the MINORS scores ranged from 13 to 15, indicating less risk of bias assessment for the articles in the included studies. The details of the included studies are provided in **Table 1**.

**Table 1:** Details of the included studies.

First author/s, year	Country	Patients, *n*	Age (years)	Follow-up (months)	Tumor stage				Quality evaluation
					Tis	T0	T1	T2	
Allaix et al. (2009) (29)	Italy	70	65.3 ± 11.6	82 ± 39	0	0	38	32	14
Baatrup et al. (2008)	Denmark	119	77	No	0	0	72	47	13
Borschitz et al. (2007) (25)	Germany	20	71 ± 10	45 ± 44	0	0	0	20	13
Bretagnol et al. (2007) (26)	UK	48	69	34	0	0	31	17	14
Floyd et al. (2005) (27)	America	53	65.6	34.1	0	0	53	0	13
Gao et al. (2014) (28)	China	36	49.4 ± 11.6	19.8 ± 3.4	9	0	27	0	14
Guerrieri et al. (2004) (29)	Italy	114	64.8	46	0	18	37	59	15
Hart et al. (2023) (30)	New Zealand	24	75.9	29.5	0	0	19	5	14
Jeong et al. (2009) (31)	Korea	25	52	37	0	2	17	6	14
Jones et al. (2018) (32)	UK	70	70	39.6	0	0	70	0	13
Kanehira et al. (2013) (33)	Japan	153	64.7	46.4	0	115	38	0	14
Khoury et al. (2022) (34)	Israel	43	69 ± 9	32	0	0	43	0	13
Lezoche et al. (2011) (35)	Italy	135	63	97	0	24	66	45	15
Maslekar et al. (2006) (36)	UK	49	74.3	40	0	0	27	22	14
Mentges et al. (1996) (37)	Germany	83	68.9	24	0	0	56	27	14
Morino et al. (2011) (38)	Italy	91	68.4	54.2	0	0	48	43	14
Osman et al. (2016) (39)	UK	38	71 ± 10	13 ± 11	0	1	19	18	15
O’Neill et al. (2017) (40)	America	92	68.1 ± 11.4	55.2	0	8	54	30	15
Perez et al. (2012) (41)	Brazil	27	58.3 ± 10.9	15	0	3	6	18	15
Ramirez et al. (2011) (42)	Spain	81	69	71	0	0	59	22	14
Ren et al. (2021) (43)	China	41	73.9 ± 3.3	17.4 ± 3.1	0	12	28	1	15
Samalavicius et al. (2015) (44)	Lithuania	20	71	19.7	0	0	14	6	14
Serra-Aracil et al. (2008) (45)	Spain	49	66	59	22	0	16	11	15
Smart et al. (2016) (46)	UK	61	75	13	0	20	23	18	15
Stipa et al. (2012) (47)	Italy	124	67	85	0	0	86	38	14
Sun et al. (2014) (48)	China	86	55.9	37.6	26	0	42	18	15
Sun et al. (2016) (49)	China	64	57.3 ± 8.6	20	32	0	22	10	15
Tsai et al. (2009) (50)	America	84	66.6 ± 1.9	49.5	0	0	58	26	14
Whitehouse et al. (2007) (51)	UK	32	75	34	0	0	23	9	14
Xia et al. (2010) (52)	China	84	63 ± 9	26	0	60	19	5	14
Xu et al. (2020) (53)	China	62	62	52.5	0	0	36	26	14
Yu et al. (2013) (54)	China	50	49	31.6	0	0	50	0	13
Zhuang et al. (2013) (55)	China	32	57.6 ± 8.7	25	16	0	11	5	15

### 3.3 Survival rate

**Figure 2:**
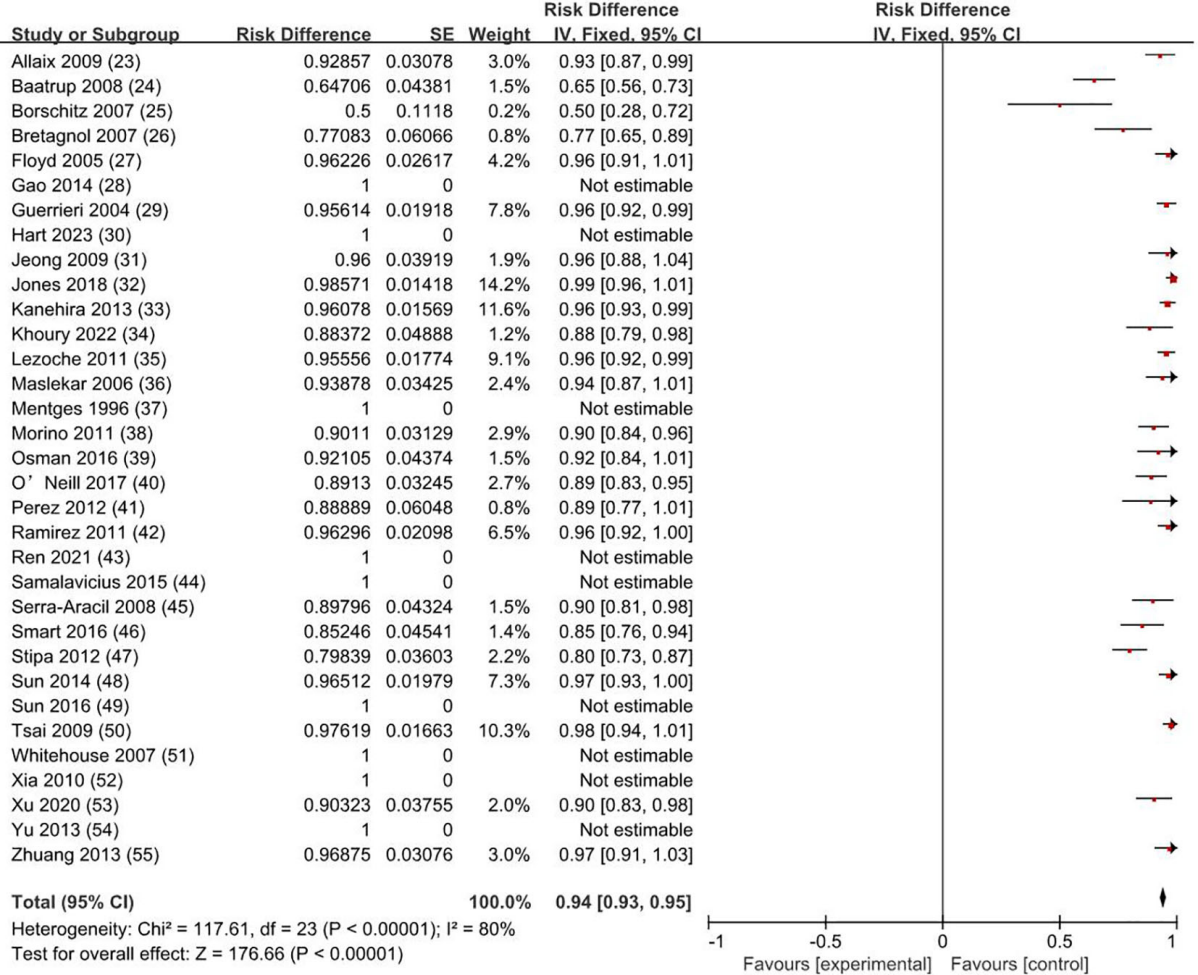
Overall survival rate. SE, standard error; CI, confidence interval.

**Figure 3:**
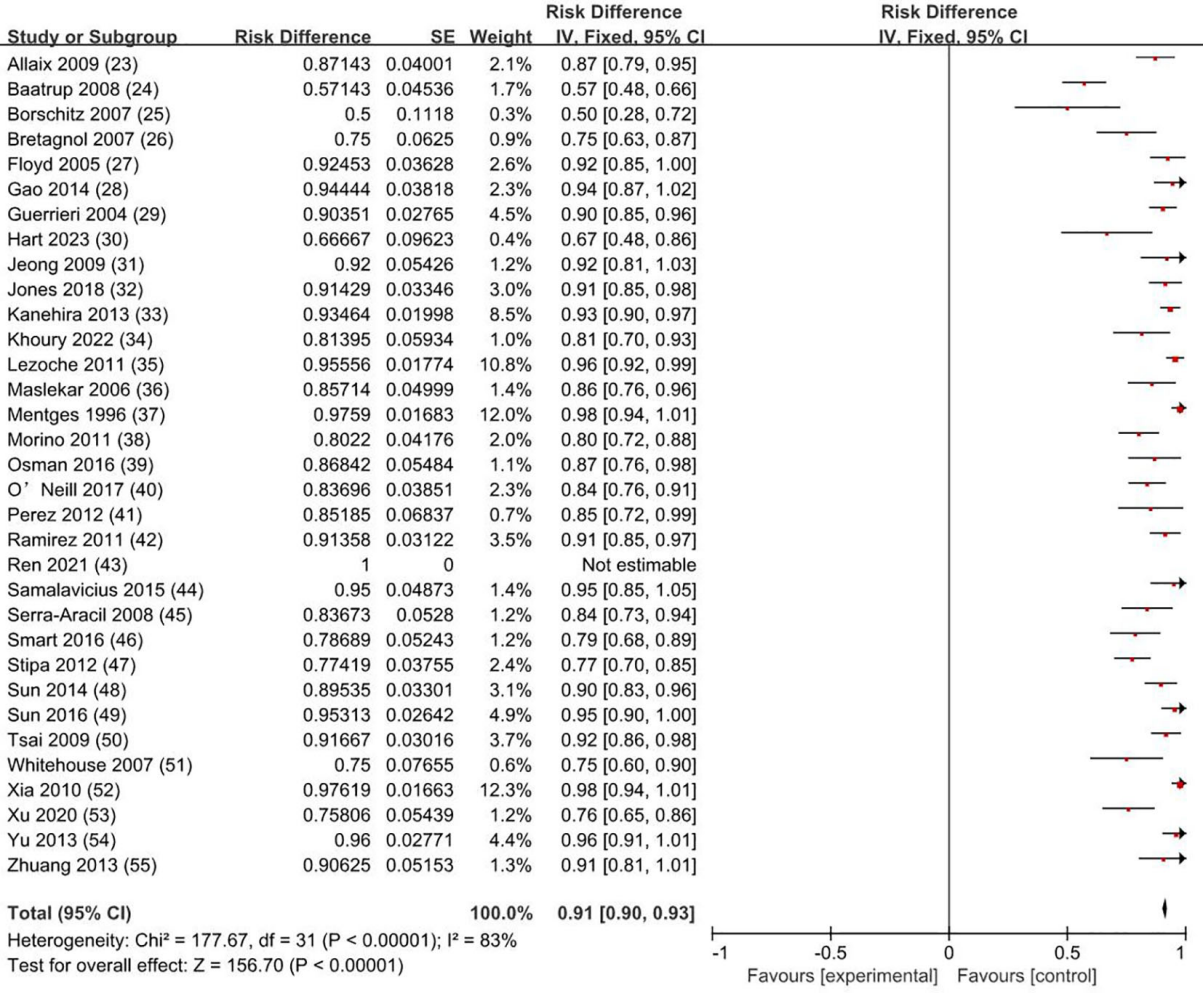
Disease-free survival rate. SE, standard error; CI, confidence interval.

**Figure 4:**
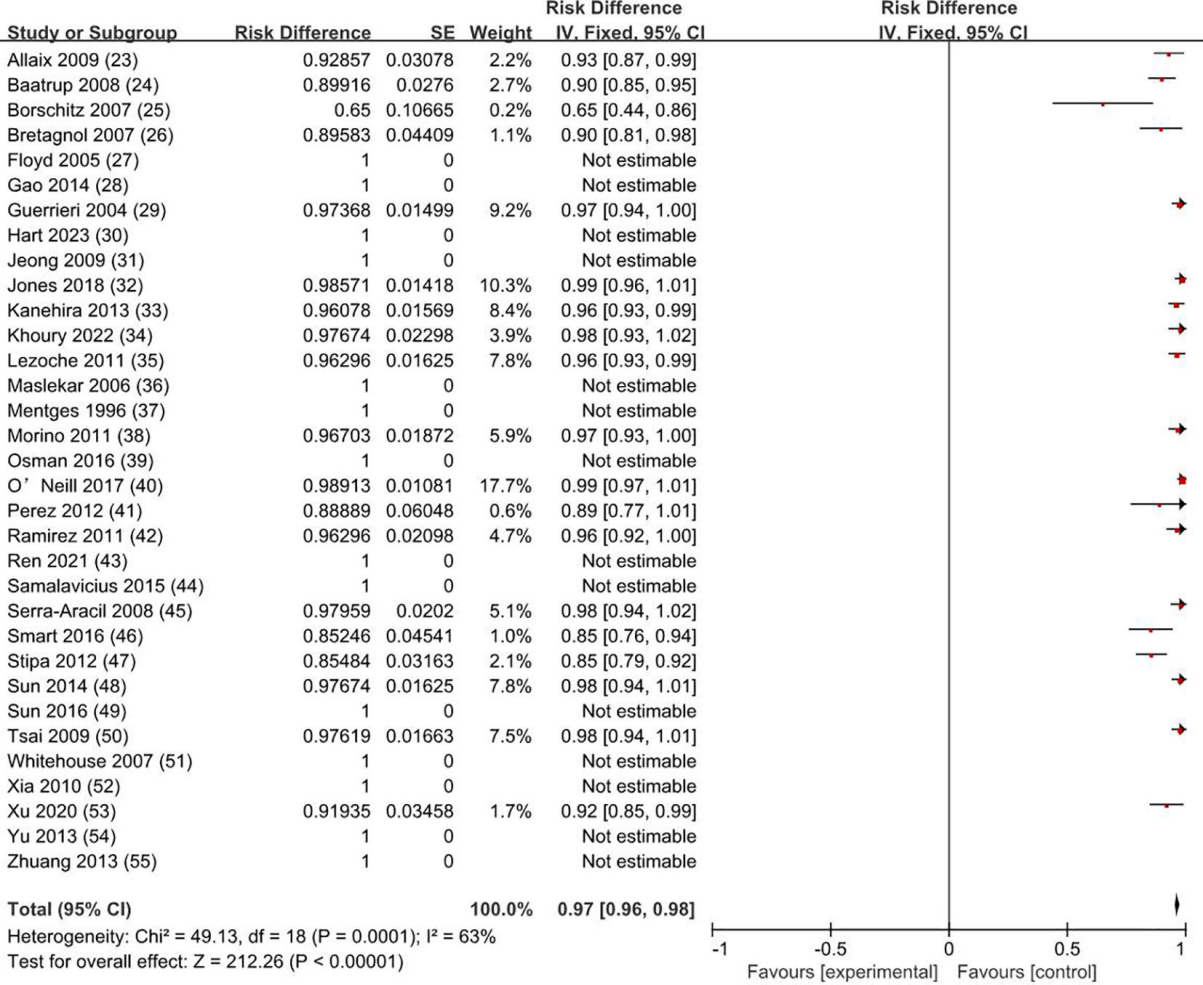
Disease-specific survival rate. SE, standard error; CI, confidence interval.

The overall survival rate was 100% for T0 stage, 98.1% for Tis stage, and 80.2% for early stage rectal cancer patients (83.9% for T1 and 72.4% for T2). The weighted average overall survival rate was 94% (RD = 0.94; 95% CI = 0.93–0.95; *I* ^2^ = 80%; *P* < 0.00001) for all stage patients (**Figure 2**); the weighted average disease-free survival was 91% (RD = 0.91; 95% CI = 0.90–0.93; *I* ^2^ = 83%; *P* < 0.00001) (**Figure 3**); and disease-specific survival was 97% (RD = 0.97; 95% CI = 0.96–0.98; *I* ^2^ = 63%; *P* < 0.00001) (**Figure 4**).

### 3.4 Recurrence rate

**Figure 5:**
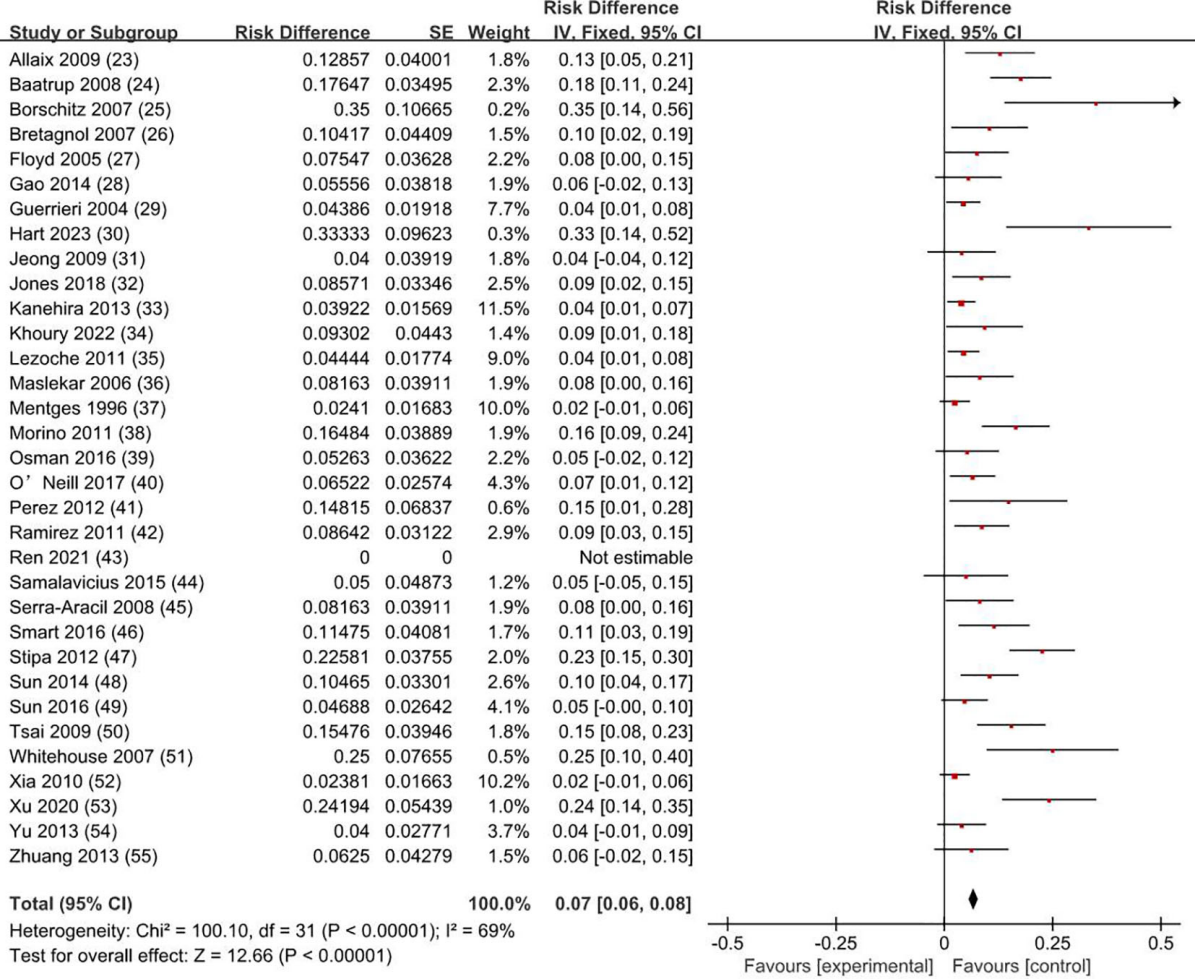
Recurrence rate. SE, standard error; CI, confidence interval.

Our meta-analysis showed that the recurrence rate was 0.5% for T0 stage, 1.9% for Tis stage, and 11.9% for early stage rectal cancer patients (8.1% for T1 and 19.7% for T2). The weighted average recurrence was 7% (RD = 0.07; 95% CI = 0.06–0.08; *I* ^2^ = 69%; *P* < 0.00001) for all stage patients (**Figure 5**).

### 3.5 Complications rate and types

**Figure 6:**
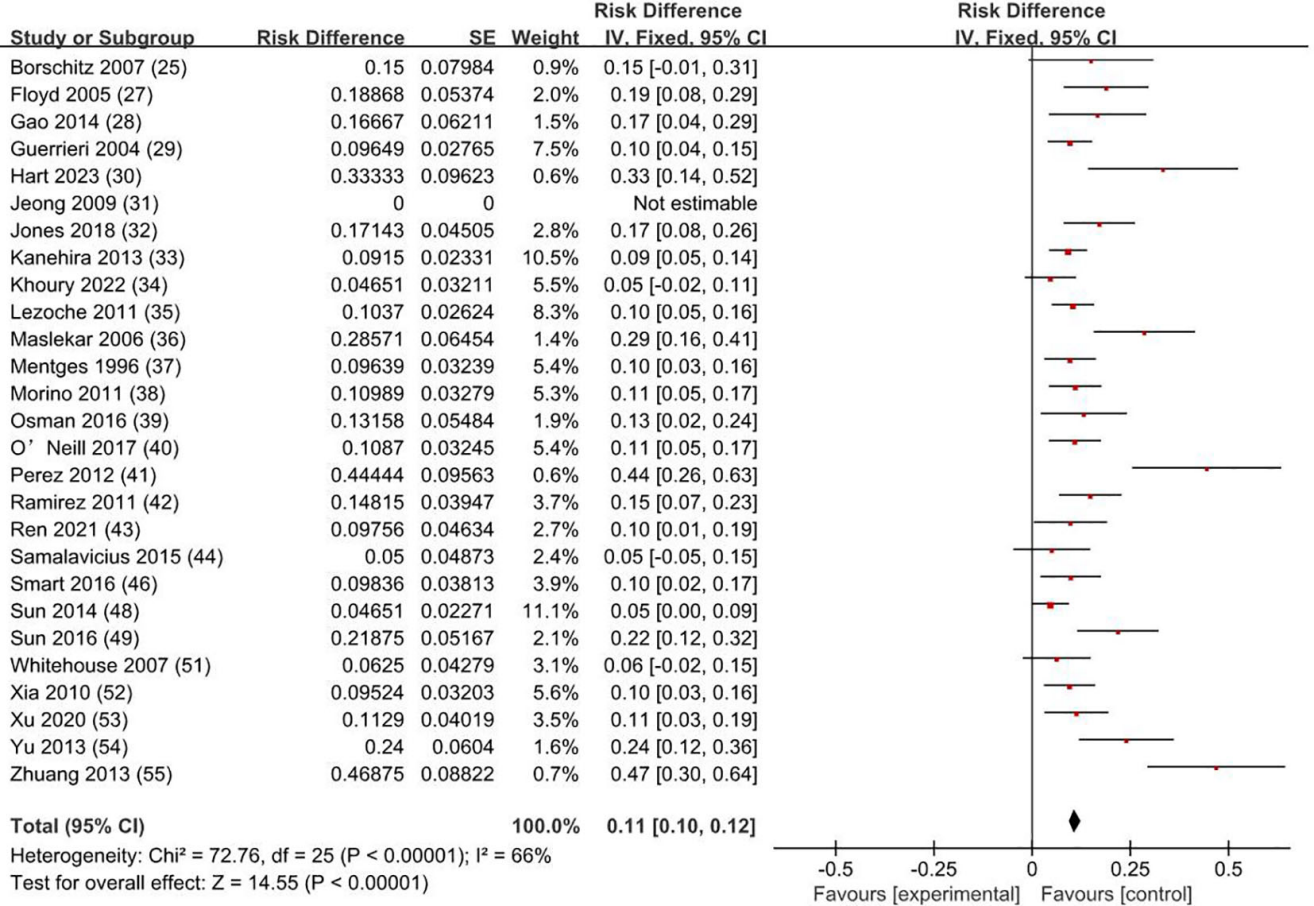
Complication rate. SE, standard error; CI, confidence interval.

**Figure 7:**
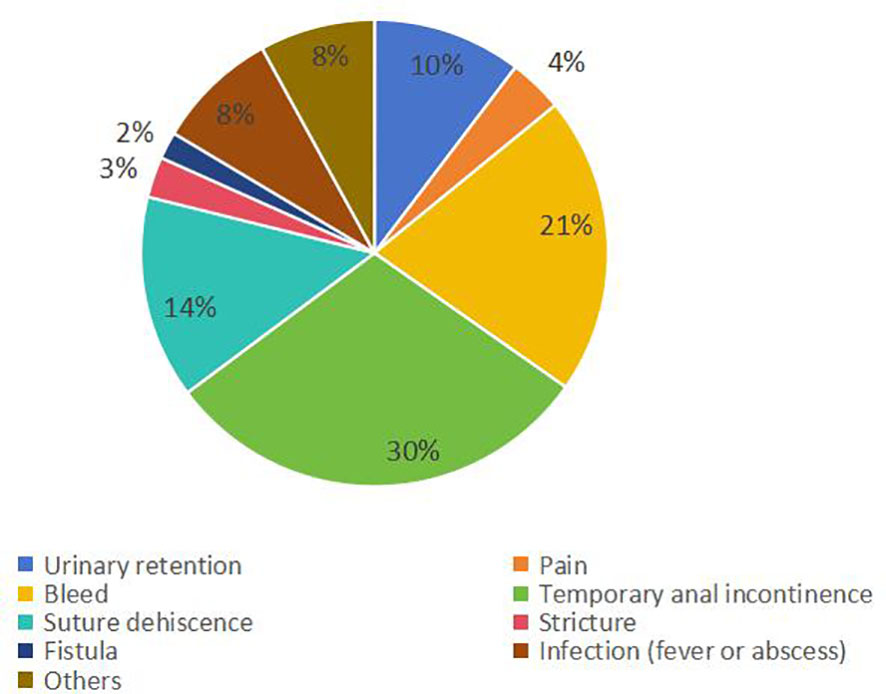
Type and proportion of complications.

The weighted average complication rate was 11% (RD = 0.11; 95% CI = 0.10–0.12; *I* ^2^ = 66%; *P* < 0.00001) (**Figure 6**) for all stage patients, with Clavien-Dindo grade I accounting for 77.7%, Clavien-Dindo grade II accounting for 8%, and Clavien-Dindo grade III accounting for 14.3%. The most common postoperative complication was temporary anal incontinence in 64 patients (30%), followed by bleeding in 44 patients (21%) and dehiscence of sutures in 30 patients (14%). Specific types of complications are shown in **Figure 7**.

## 4 Discussion

Early stage rectal cancer refers to the rectal infiltrating adenocarcinoma that only invaded the submucosa or muscularis propria, with no lymph node metastasis or distant metastasis (T1N0M0, T2N0M0), accounting for about 11% of rectal cancer. T0 refers to the absence of evidence of a primary tumor, and Tis refers to carcinoma *in situ* (the tumor invades the proper muc layer but does not penetrate the muscularis mucosae) (56). Salinas HM et al. found that 89% of T1 patients and 72% of T2 patients experienced unnecessary radical resection (57). TEM combines endoscopic techniques and minimally invasive techniques, with the advantages of less trauma, sufficient visual field exposure, and precise surgical resection (58). TEM removes only the local tumor tissue and cannot clear occult metastatic lymph nodes, while the probability of lymph node metastasis was considered to be 0%–15% in T1 and 16%–28% in T2 (59). The results of the clinical study by Stornes T et al. showed that the 5-year local recurrence rate of TEM patients was 14.5% (T1), and 11.4% (T2), which was significantly higher than the 1.4% (T1) and 4.4% (T2) of total mesorectal excision (TME) (60). Therefore, although many physicians advocate the use of TEM for early rectal cancer, its safety and efficacy remain controversial.

The results of our analysis showed that the recurrence rate was 0.5% for T0 stage, 1.9% for Tis stage, and 11.9% for early stage rectal cancer patients (8.1% for T1 and 19.7% for T2). The weighted average recurrence rate was 7% for all stage patients. Junginger T et al. believe that local tumor recurrence after rectal cancer surgery is related to residual postoperative tumor tissue, and they think that the integrity of tumor resection and negative resection margin are key factors in reducing the local recurrence rate (61). Chen YY et al. suggested that local tumor recurrence after rectal cancer surgery is related to lymphatic metastasis due to incomplete regional lymph node resection, and they think that potential lymph node metastasis in the mesentery is the main cause of local recurrence (22). Weiser M R agrees with Chen YY et al. and believes that preoperative classification of lymph node metastasis is a key factor in patient selection of treatment (62). This may explain why the results of this study show that the recurrence rate of T2 stage rectal cancer is significantly higher than that of T1 stage. Morino M et al. believe that tumor size is also one of the reasons for the recurrence of rectal cancer after surgery; the larger the tumor, the higher the risk of postoperative recurrence (38). McCloud et al. found that the recurrence rate of rectal tumors with >5cm was significantly higher than that of smaller tumors (25.9% vs. 8.9%) (63). In addition, some studies believe that the degree of tumor differentiation and nerve vascular invasion around the tumor is associated with postoperative recurrence (61, 64). Local resection is a minimum resection of the rectal wall under the premise of ensuring radical treatment. The traditional transanal local resection after the surgical margin positive rate is 27%, and the local recurrence rate is as high as 39% (65). By combining minimally invasive surgical techniques with endoscopic microscopy, TEM can better expose the surgical field, achieve complete resection of the tumor, and reduce the positive margin rate. Currently, patients with rectal masses that are assessed as T0 or Tis stage after thorough preoperative evaluation, or T1 stage rectal cancer with tumor invasion <30% of the intestinal circumference, tumor size <3 cm, good differentiation, first layer of the submucosa, no vascular or neural invasion, and exclusion of lymph node metastasis and distant metastasis, are considered to have low-risk features for local recurrence (61, 66). Unfortunately, few of the articles we included (26, 32, 33, 38, 39, 54) distinguished and analyzed the pathological factors and depth of submucosal invasion associated with poor prognosis in T1 rectal cancer. Among all the patients with T1 rectal cancer who had a recurrence and whose tumor invasion depth was recorded, the Sm2+Sm3 recurrence rate was 83.3%. The study by Kapiteijn E et al. reported that the local recurrence rate after TEM in patients with T1-2N0 rectal cancer was 0.7% (67). In our analysis, the recurrence rate after TEM for T2 rectal cancer was 19.7%, which was higher than the 11.4% reported by Stornes T et al., and much higher than the 4.4% recurrence rate after TME (60). Such a high recurrence is unacceptable therefore, for such patients, TME is more frequently recommended.

Our study showed that the overall survival rate was 100% for T0 stage, 98.1% for Tis stage, and 80.2% for early stage rectal cancer patients (83.9% for T1 and 72.4% for T2). The weighted overall survival rate was 94% for all stage patients, the weighted disease-free survival rate was 91%, and the disease-specific survival rate was 97%. How to improve the survival rate of cancer patients and avoid their death has always been one of the important purposes pursued by clinicians. Patients with early rectal cancer have a lower risk of lymph node metastasis and distant metastasis, and whether it is necessary to carry on radical resection is still quite controversial. Through complete resection of local tumor tissue, TEM is now favored by the majority of clinicians and early rectal cancer patients. Zaheer et al. showed that the overall survival after radical resection of stage I tumors was 85% (68). Hazard et al. showed that the 5-year disease-specific survival rate of T1 and T2 after radical resection was 97% and 95% (69). The results of this study showed that disease-specific survival after TEM was comparable to radical resection without significant differences. Furthermore, there was no significant difference in disease-free survival between local excision and radical resection, as shown by Tan S et al. (70).

Middleton PF et al. reported a complication rate of 0% to 28% after TEM (71). The results of this study showed that the weighted complication rate was 11% for all patients, with Clavien-Dindo grade I accounting for 77.7%, Clavien-Dindo grade II accounting for 8%, and Clavien-Dindo grade III accounting for 14.3%. Simon P Bach et al. reported that common complications of radical resection include genitourinary function impairment and irregular defecation (72). Unlike radical resection, TEM, as a surgical method of local resection, combining the advantages of minimally invasive surgery and endoscopic surgery, can avoid autonomic nerve injury in the pelvic cavity, thus avoiding injury to patients’ urogenital function (73). Our study showed that most patients have Clavien-Dindo I postoperative complications, which generally do not require special treatment, with only a minority of Clavien-Dindo II–III patients needing interventions such as blood transfusions or surgery. The most common complication after TEM was temporary anal incontinence, accounting for 3.8% of the total population (30% of the complication). Cataldo PA et al. believe that the reason may be the thicker diameter of the endoscope used by TEM (4 cm), which leads to overstretching injury of the anal sphincter, resulting in the occurrence of temporary anal incontinence, but the symptoms will disappear within three months (74). Our study showed that the bleeding complications after TEM represented 2.6% of the total population (21% of the complications). The reason may come from the surgical wound or anal enlargement causing anal skin tear and internal hemorrhoid bleeding, which is usually controllable. Furthermore, our study showed that 1.8% of patients had postoperative suture dehiscence (14% of the complication), and the possible reason is the large wound surface and the excessive postoperative suture tension. Wei Li et al. by meta-analysis showed that the complication rate after TEM is lower than after radical resection (75).

Of course, our study also has some limitations. First, some of the included literature are retrospective studies, and the authenticity and accuracy of the collected data were low; second, due to differences in inclusion criteria, there is a certain degree of heterogeneity; third, the different follow-up times of patients in different literature may affect the postoperative recurrence rate and survival outcomes. Therefore, more prospective studies with uniform criteria are needed.

## 5 Conclusions

In conclusion, limited evidence indicates that TEM, as an alternative to radical resection of rectal cancer, has a low rate of postoperative complications in treat of T1 rectal cancer, T0 stage and Tis stage tumors, consistent with the minimally invasive surgical treatment philosophy. TEM has a high-postoperative survival rate and a low recurrence rate, consistent with the management goal of rectal tumor patients. For the treatment of T2 rectal cancer, the overall survival rate is low and the recurrence rate is high, it is not recommended to use TEM alone as a radical treatment.

## Data Availability

The original contributions presented in the study are included in the article/supplementary material. Further inquiries can be directed to the corresponding author.
